# *Bartonella quintana* lipopolysaccharide (LPS): structure and characteristics of a potent TLR4 antagonist for *in-vitro* and *in-vivo* applications

**DOI:** 10.1038/srep34221

**Published:** 2016-09-27

**Authors:** Gosia Malgorzata-Miller, Lena Heinbockel, Klaus Brandenburg, Jos W. M. van der Meer, Mihai G. Netea, Leo A. B. Joosten

**Affiliations:** 1 Department of Internal Radboud University Medical Center, Nijmegen, 6500HB, The Netherlands; 2 Radboud Institute for Molecular Life Sciences, Radboud University Medical Center, Nijmegen, The Netherlands; 3 Division of Biophysics, Research Center Borstel, Leibniz-Center for Medicine and Biosciences, Borstel, Germany

## Abstract

The pattern recognition receptor TLR4 is well known as a crucial receptor during infection and inflammation. Several TLR4 antagonists have been reported to inhibit the function of TLR4. Both natural occurring antagonists, lipopolysaccharide (LPS) from Gram-negative bacteria as well as synthetic compounds based on the lipid A structure of LPS have been described as potent inhibitors of TLR4. Here, we have examined the characteristics of a natural TLR4 antagonist, isolated from *Bartonella quintana* bacterium by elucidating its chemical primary structure. We have found that this TLR4 antagonist is actually a lipooligosaccharide (LOS) instead of a LPS, and that it acts very effective, with a high inhibitory activity against triggering by the LPS-TLR4 system in the presence of a potent TLR4 agonist (*E. coli* LPS). Furthermore, we demonstrate that *B. quintana* LPS is not inactivated by polymyxin B, a classical cyclic cationic polypeptide antibiotic that bind the lipid A part of LPS, such as *E. coli* LPS. Using a murine LPS/D-galactosamine endotoxaemia model we showed that treatment with *B. quintana* LPS could improve the survival rate significantly. Since endogenous TLR4 ligands have been associated with several inflammatory- and immune-diseases, *B. quintana* LPS might be a novel therapeutic strategy for TLR4-driven pathologies.

The innate immune system is able to recognize multiple microbial components, including those of Gram-positive and Gram-negative bacteria, fungi and viruses. Recognition of Gram-negative bacteria mainly occurs via lipopolysaccharide (LPS), one of the major components of the outer membrane of these bacteria. The lipid A moiety of LPS interacts with a membrane receptor complex containing Toll-like receptor 4 (TLR4), MD-2, and CD14 and thereby induces proinflammatory cytokines, chemokines, and adhesion molecules. Together, these mediators may evoke the clinical signs of bacteria-induced sepsis^1^,^2^,^3^. Apart from microbial ligands, TLR4 is able to recognize endogenous ligands, such as breakdown products of extracellular matrix, alarmins, and intracellular proteins^4^,^5^,^6^,^7^,^8^. It has been suggested that these endogenous TLR4 ligands are important for the vicious loop during chronic inflammation^9^,^10^,^11^ and hence TLR4 is linked to the pathogenesis of several autoimmune diseases including rheumatoid arthritis, systemic lupus erythematosus, systemic sclerosis, Sjogren’s syndrome, psoriasis, multiple sclerosis, Atherosclerosis, and autoimmune diabetes^12^,^13^,^14^. The inhibition of TLR-4 activation has been investigated as potential anti-inflammatory therapy for many inflammatory diseases, including rheumatoid arthritis^15^.

*Bartonella quintana* is a louse-borne Gram-negative pathogen, which has been originally described during World War I as the causative microbe of trench fever, a disease associated with recurrent fever and headaches. *B. quintana* bacteria colonize the louse alimentary tract enabling a single louse to infect multiple humans^16^,^17^,^18^,^19^,^20^. After introduction into the human host, *B. quintana* can persist in the normally sterile bloodstream for weeks or month. This remarkable, prolonged persistence in the host bloodstream demonstrates the ability of *B. quintana* to avoid clearance by the host immune defense^21^. Furthermore, it has been observed that patients with *B. quintana* bacteremia do not show the classical sepsis syndrome. As an explanation for this phenomenon, overproduction of the anti-inflammatory cytokine interleukin-10 (IL-10) and an attenuated inflammatory cytokine profile during *B. quintana* bacteremia have been proposed^22^. We have previously described the anti-inflammatory effect of *B. quintana* LPS. The molecule blocks TLR4 activation and it has been shown that in several *in vitro* and *in vivo* models *B. quintana* LPS can be used as a potential therapeutic agent for the treatment of rheumatoid arthritis, ventilation-induced lung injury (VILI), atherosclerosis and other autoinflammatory diseases^15^,^23^,^24^,^25^. In the present study, we investigated the properties of *B. quintana* LPS in more details, in terms of induction of cytokines (pro- and anti-inflammatory), the potency to block TLR4, the kinetics of TLR4 antagonism and interaction with TLRs and other species of LPS.

## Results

### *Bartonella quintana* LPS does not induce production of pro- or anti-inflammatory cytokines

**Figure 1:**
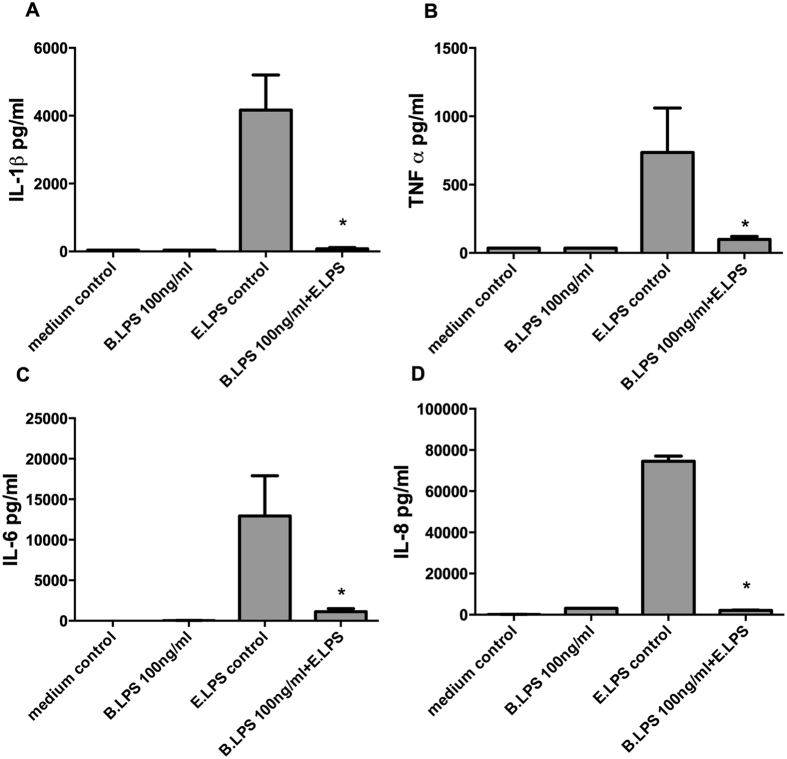
No agonistic effect of *B. quintana* LPS on human PBMCs. Human PBMCs were isolated from healthy subjects, using a standard protocol. PBMCs were pre-incubated with 100 ng/ml *B. quintana* LPS for 2 h, and thereafter 10 ng/ml purified *E. coli* LPS was added as indicated in the graph. Cytokines were determined after 24 h of culture by ELISA, IL-1β (**A**), TNF-α (**B**), IL-6 (**C**) and IL-8 (**D**). PBMCs of 6 healthy donors were examined. *P < 0.001, two-sided Mann-Whitney *U* test.

**Figure 2:**
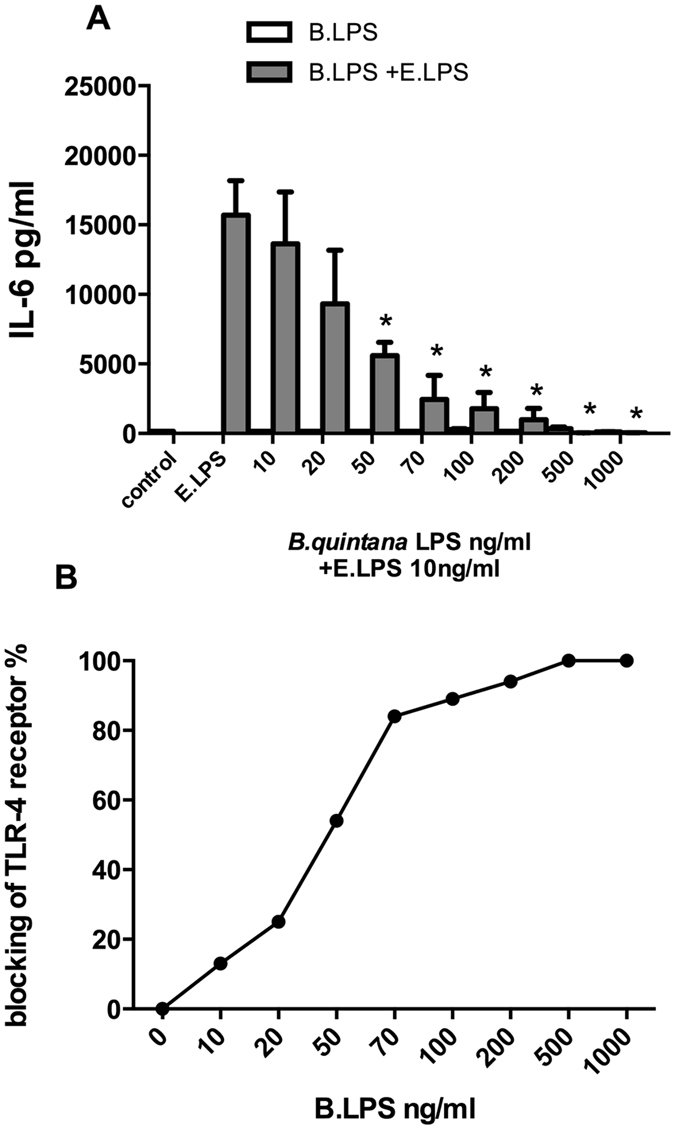
Dose-response of the TLR4 antagonist *B. quintana* LPS. Human PBMCs were isolated from healthy subjects, using a standard protocol. PBMCs were pre-incubated with a dose-range of *B. quintana* LPS (10–1000 ng/ml) for 2 h. Thereafter, 10 ng/ml *E. coli* LPS was added and the PBMCs were cultured for another 24 h. IL-6 was determined by using ELISA (**A**). (**B**) Percentage inhibition was calculated using the IL-6 concentration of *E. coli* LPS exposure as 100%. PBMCs of 6 subjects were used in this experiment. IC50 was 37.04 ng/ml. *P < 0.001, two-sided Mann-Whitney *U* test.

The first sets of experiments were designed to investigate whether exposure to *B. quintana* LPS results in the induction of pro- or anti-inflammatory cytokines by human PBMCs. As shown in Fig. 1, *B. quintana* LPS itself does not induce the production IL-1β, TNF-α, IL-6 or IL-8. In addition, exposure for 24 h with *B. quintana* LPS did not result in the production or release of IL-1Ra, or IL-10 by human primary PBMCs (data not shown). However, *B. quintana* LPS efficiently blocks production of IL-1β, TNF-α, IL-6, IL-8 or after stimulation of human PBMCs with *E. coli* LPS, indicating the potency of *B. quintana* LPS as TLR4 antagonist. In addition, we performed dose-response experiments to examine the IC50 of *B. quintana* LPS for the standard dose of 10 ml *E. coli* LPS. Figure 2A shows that already 20 ng of *B. quintana* LPS reduced the IL-6 production. At higher concentrations of *B. quintana* LPS, 5-fold or higher, a strong suppression of the IL-6 production was stated. Figure 2B demonstrated that *B. quintana* LPS revealed to have an IC50 of 37.04 ng/ml at a dose of 10 ng/ml ultra pure *E. coli* LPS. These data confirmed that LPS isolated of *B. quintana* is a very potent TLR4 antagonist at a low concentration^23^.

### Prolonged blocking of TLR4 by *B. quintana* LPS

**Figure 3:**
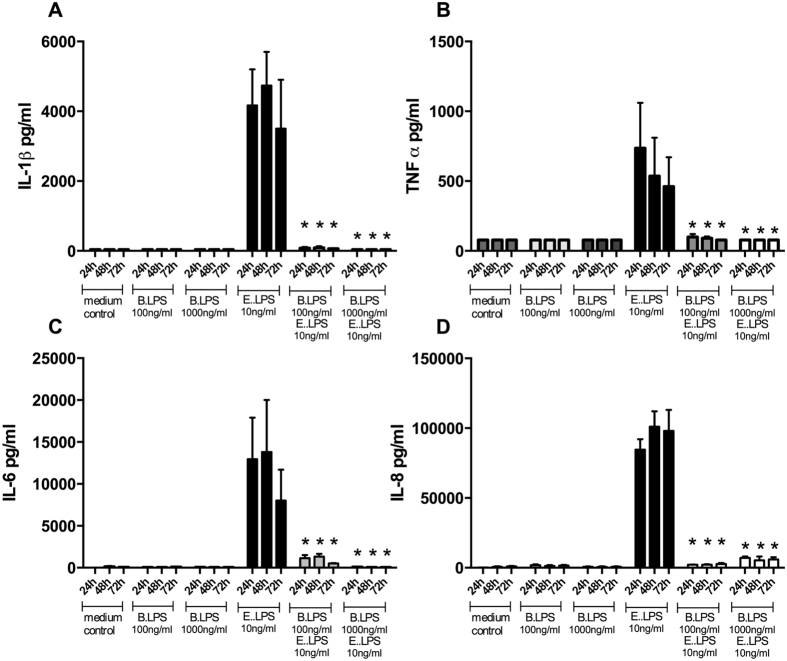
Time course of TLR4 inhibition in the presence of *B. quintana* LPS. Human PBMCs were isolated from healthy subjects, using a standard protocol. PBMCs were pre-incubated with 100 ng/ml or 1000 ng/ml *B. quintana* LPS for 2 h. Thereafter 10 ng/ml purified *E. coli* LPS was added as indicated in the graph. Cytokines were determined after 24 h, 48 h or 72 h of culture by standard ELISA for IL-1β (**A**), TNF-α (**B**), IL-6 (**C**) and IL-8 (**D**). PBMCs of 6 healthy donors were examined. *P < 0.001, two-sided Mann-Whitney *U* test.

**Figure 4:**
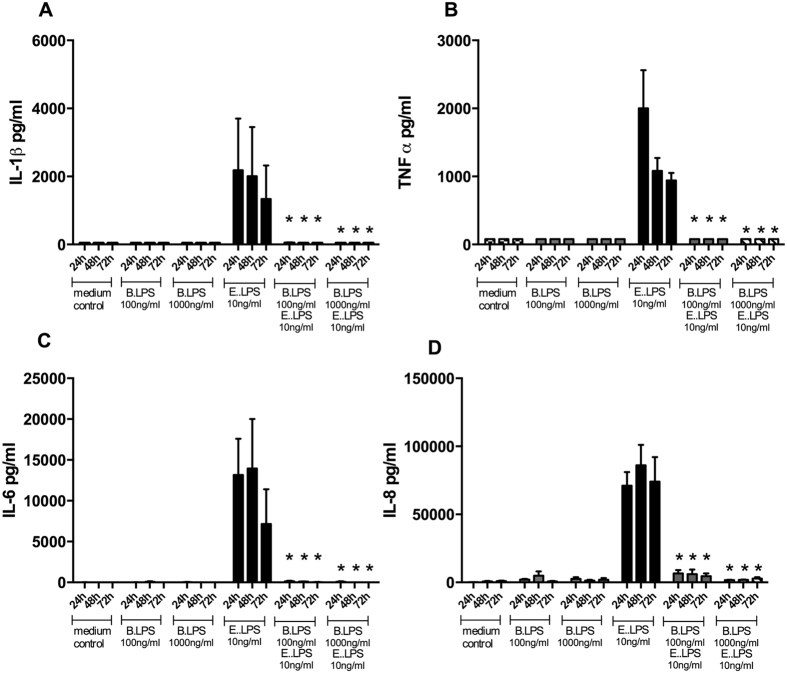
Time course of the TLR4 inhibitor after removal of the *B. quintana* LPS. Human PBMCs were isolated from healthy subjects, using a standard protocol. PBMCs were pre-incubated with 100 ng/ml or 1000 ng/ml *B. quintana* LPS for 2 h. Thereafter the PBMCs were extensively washed (3 times) to removed the non-bound *B. quintana* LPS. After the washing step, 10 ng/ml purified *E. coli* LPS was added. Cytokines were determined after 24 h, 48 h or 72 h of culture by standard ELISA for IL-1β (**A**), TNF-α (**B**), IL-6 (**C**) and IL-8 (**D**). PBMCs of 6 healthy donors were examined. *P < 0.001, two-sided Mann-Whitney *U* test.

In order to investigate the kinetics of the bindings capacity of *B. quintana* LPS to TLR4 and whether removing of the TLR4 inhibitor has an effect on blockade TLR4 function, we pre-incubated human PBMCs with *B. quintana* LPS for 1 hour. Two different approaches were investigated. The first approach was that *B. quintana* LPS was continuously present during the exposure to *E. coli* LPS and in the second approach we removed the *B. quinta*na LPS by thorough washing (3 times). Thereafter the PBMCs were exposed to *E. coli* LPS and cells were incubated for additional 24 h, 48 h or 72 h hours. At each time point the cells were microscopically checked and the supernatant was collected to measure IL-1β, IL-6, IL-8, or TNF-α. Figure 3 shows that *B. quintana* LPS blocks the cytokine production by *E. coli* LPS at least for a period of 72 h. Cytokine production by human PBMCs is reduced for more than 90% over this exposure period, when the *B. quintana* LPS is present in the culture medium. The second approach in which *B. quintana* LPS was removed after 1 hour by repeated washing, identical effects on the neutralizing capacity of *B. quintana* LPS were seen (Fig. 4): Almost complete inhibition of the *E. coli* LPS-induced cytokine production after 72 h culture in the presence of 10 ng/ml of this classical TLR4 agonist. Thus, our data show that the blocking of TLR4 by *B. quintana* LPS is strong and stable for at least 72 hours.

### Rapidity of binding of *B. quintana* LPS to TLR4

**Figure 5:**
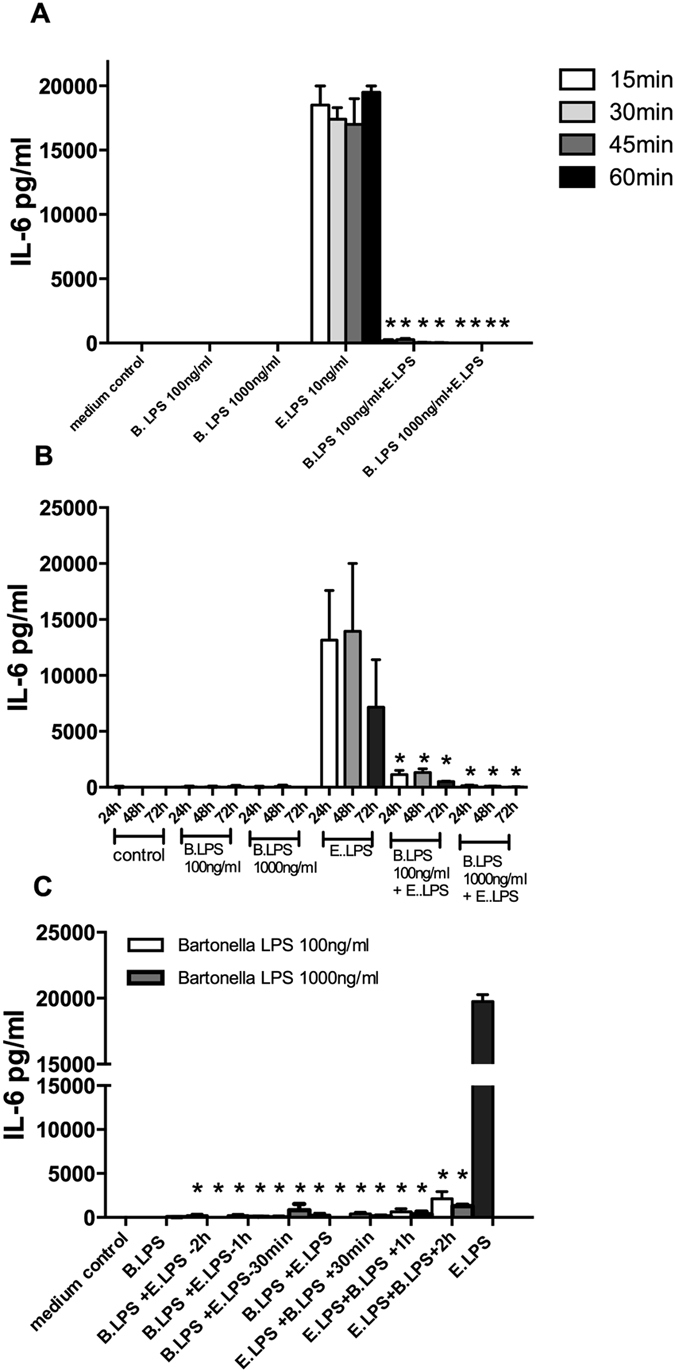
Kinetics of the *B. quintana LPS* to block the TLR4. Human PBMCs were isolated from healthy subjects, using a standard protocol. (**A**) PBMCs were pre-incubated with 100 ng/ml or 1000 ng/ml *B. quintana* LPS for different times before *E. coli* LPS was added to the culture medium. After 1 hour, 45, 30 and 15 minutes PBMC were exposed to 10 ng/ml *E. coli* LPS for 24 h. (**B**) *B. quintana* LPS was added together with *E. coli* LPS, thereafter the PBMCs were cultured for additional 24 h, 48 h or 72 h. C, *B. quintana* LPS was added in a range before (−2 h) and after (+2 h) the cells were exposed to 10 ng/ml *E. coli* LPS. IL-6 was determined by using ELISA. PBMCs of 4 subjects were used in this experiment. *P < 0.001, two-sided Mann-Whitney *U* test.

To explore the capacity of *B. quintana* LPS to bind the TLR4 over time, we examined the minimal pre-incubation time that allows *B. quintana* LPS to block TLR4 completely. Therefore we pre-incubated PBMCs with 100 ng/ml and 1000 ng/ml *B. quintana* LPS for 15, 30, 45 or 60 minutes and added 10 ng/ml *E. coli* LPS. After 24 h the IL-6 concentrations were determined in the supernatants. Figure 5A shows that 15 minutes’ pre-incubation is sufficient for 100 ng/ml of *B. quintana* LPS to block TLR4 receptor. The rapid binding of *B. quintana* LPS to TLR4 indicates that this TLR4 inhibitor is efficient.

### *B. quintana* LPS neutralizes TLR4 even in the presence of *E. coli* LPS

Since we noted that *B. quintana* LPS binds very rapidly to TLR4, we investigated the neutralizing capacity of *B. quintana* LPS when added together with *E. coli* LPS or even after addition of *E. coli* LPS to the culture medium. Figure 5B indicates that *B. quintana* LPS added together with *E. coli* LPS blocks the TLR4 receptor for at least 72 hours. IL-6 production due to 10 ng/ml *E. coli* LPS was completely suppressed by 10 times excess of *B. quintana* LPS. Remarkably, when *B. quintana* LPS was added 2 hours after the PBMC were exposed to *E. coli* LPS, we still noted a strong suppression of the IL-6 production. Figure 5C reveals that even a dose of 100 ng/ml *B. quintana* LPS (10 times excess) blocks TLR4 for 72 hours, in the presence of the TLR agonist.

### Polymyxin B does not inactivate the antagonistic effect of *B. quintana* LPS

**Figure 6:**
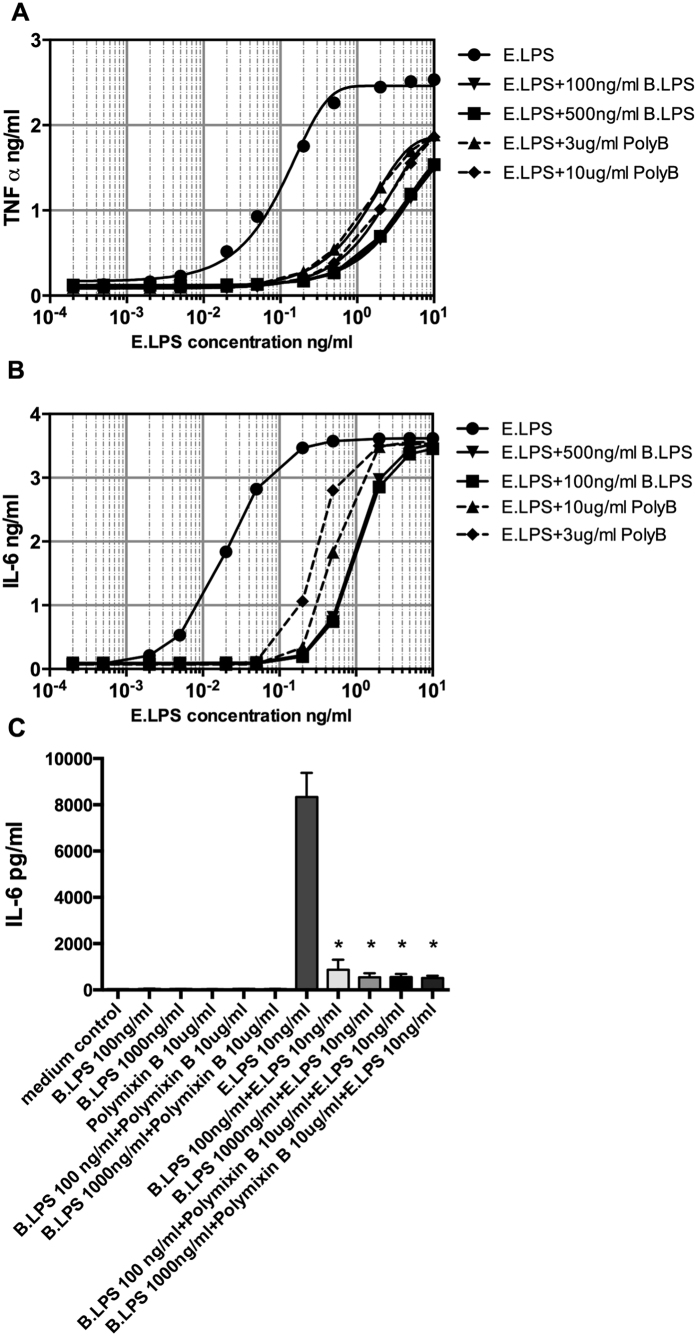
Comparison and interaction of *B. quintana* LPS with polymixin B. Human PBMCs were isolated from healthy subjects, using a standard protocol. (**A**,**B**) PMBCs were exposed to a dose range of *E. coli* LPS (0.0001 to 10 ng/ml) in the presence or absence of *B. quintana* LPS (100 and 500 ng/ml) or polymixin (**B**) (3 and 10 μg/ml). TNF-α and IL-6 were determined with ELISA. (**C**) *B. quintana* LPS (100 ng/ml or 1000 ng/ml) was pre-incubated with 10 μg/ml polymyxin B for 2 h. Thereafter control medium, *B. quintana* LPS, polymyxin (**B**) *B. quintana* LPS + polymyxin B were added to the PBMCs for 2 h and thereafter the cells were washed 3 times with warm RPMI 1640 medium. After washing, RPMI 1640 medium or E. coli LPS (10 ng/ml) was added and the PBMCs were incubated for 24 h. IL-6 was determined by using ELISA. PBMCs of 4 subjects were used in this experiment. *P < 0.001, two-sided Mann-Whitney *U* test.

It is well known that polymyxin B binds to LPS from several Gram-negative microorganisms and neutralizes the activity. First, we compared *B. quintana* LPS with polymyxin B to analyze the difference in the neutralizing capacity. Figure 6A,B showed that *B. quintana* LPS is far more potent to inhibit *E. coli* LPS mediated TNF-α or IL-6 production by human PBMCs. A concentration of 100 ng/ml *B. quintana* LPS was equally potent as 10 μg/ml polymyxin B to block 1 or 10 ng/ml of *E. coli* LPS. Thereafter, we investigated whether polymyxin B was able to bind and inactivate *B. quintana LPS*. Figure 6C demonstrated that after pre-incubation of *B. quintana* LPS with 10 μg/ml polymyxin B for 2 hours, the inhibitory capacity of *B. quintana* LPS was still very high. As control, polymyxin B neutralized the *E. coli* LPS as expected.

### *B. quintana* LPS showed efficacy in *E. coli* LPS-induced murine model of endotoxaemia

**Figure 7:**
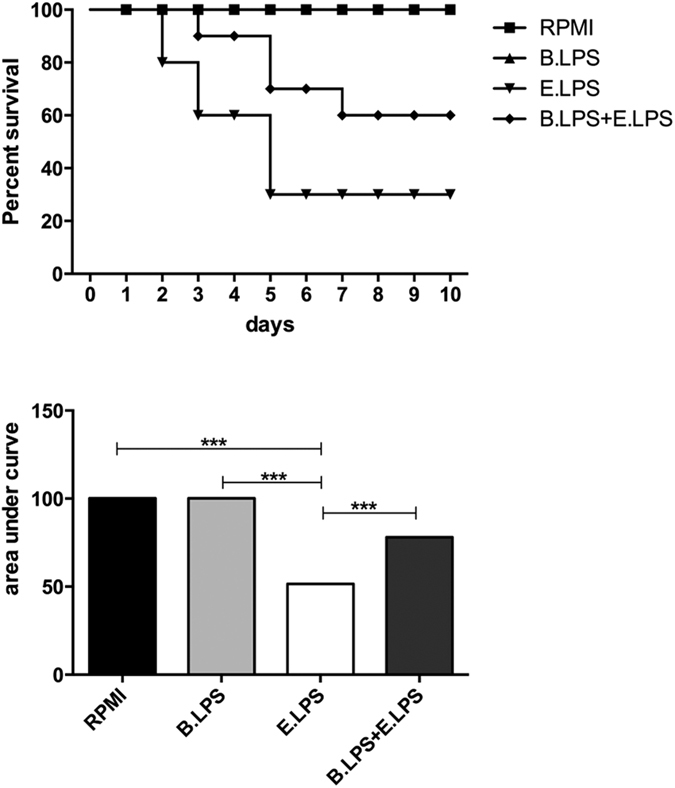
*B. quintana* LPS suppressed LPS/D-galactosamine-induced lethal endotoxaemia. C57/Bl6 mice were i.p. injected with either PBS (n = 20) or 100 μg *B. quintana* LPS (n = 20). After 30 minutes 10 mice of each group were injected i.p. with PBS and 10 mice were injected i.p. with *E. coli* LPS (1 μg) + D-galactosamine (14 mg). Survival was monitored for 10 days. (**A**) Survival rate. (**B**) Area under the curve. ***p < 0.01, one-way ANOVA test.

To explore whether *B. quintana* LPS can be used for *in vivo* studies to neutralize TLR4, we administered *B. quintana* LPS in an endotoxemia model. *B. quintana* LPS was injected 30 minutes before a sub-lethal dose of *E. coli* LPS was injected in combination with D-galactosamine. One single injection of *B. quintana* LPS revealed to be protective as can be seen in Fig. 7A,B. In contrast to the LPS/D-galactosamine group (30% survival after 10 days), *B. quintana* LPS administration had a significant higher survival rate (60%). As expected, injection of 100 μg *B. quintana* LPS alone had no detrimental effect on the survival of the mice.

### Structural analysis of *B. quintana* LPS

**Figure 8:**
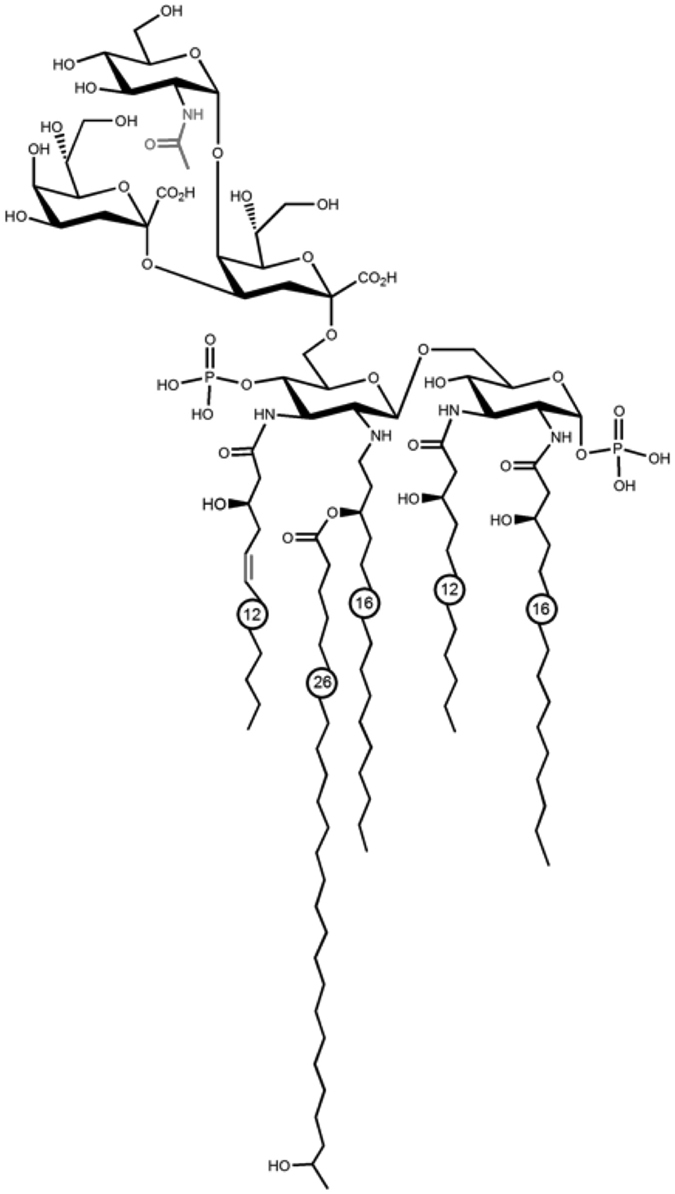
Proposed structure of *B. quintana* LPS. The primary sequence was depicted by Zähringer *et al*.^57^ for the equivalent molecule of *B. henselae*. Gas chromatographic analysis and mass spectrometry present differences of the *B. quintana* in comparison to the *B. henselae* LPS in the sugar composition and the acyl chains. The former contains a N-acetylated hexose in the outer core, while for the latter a hexose is described. Furthermore, one of the 3-OH C12 fatty acids is unsaturated in the LPS of *B. quintana*. For both substitutions the structures are not elucidated yet and the drawing shows only one of several possible positions.

In order to elucidate the structure of *B. quintana* LPS we performed gas liquid chromatography and mass spectrometry (GLC-MS) and electrospray ionization Fourier transform ion cyclotron resonance (ESI FT-ICR) analysis. The LPS of *B. quintana* appears in the MS analysis with a molecular mass of 2438,448 Dalton. By examining fragmentation products of 30 V spectra it reveals that the sugar composition of *B. quintana* LPS contains 2 Kdo, 1 HexNAc and 2 HexN. The lipids were further characterized by GLC-M and the fatty acids 25-OH C26:0, 2 3-OH C16:0, 3-OH C12:0 and 3-OH C12:1 were detected. Figure 8 showed the predicted structure of *B. quintana* LPS and it reveals that the TLR4 antagonist is actually a lipooligosaccharide (LOS).

**Figure 9:**
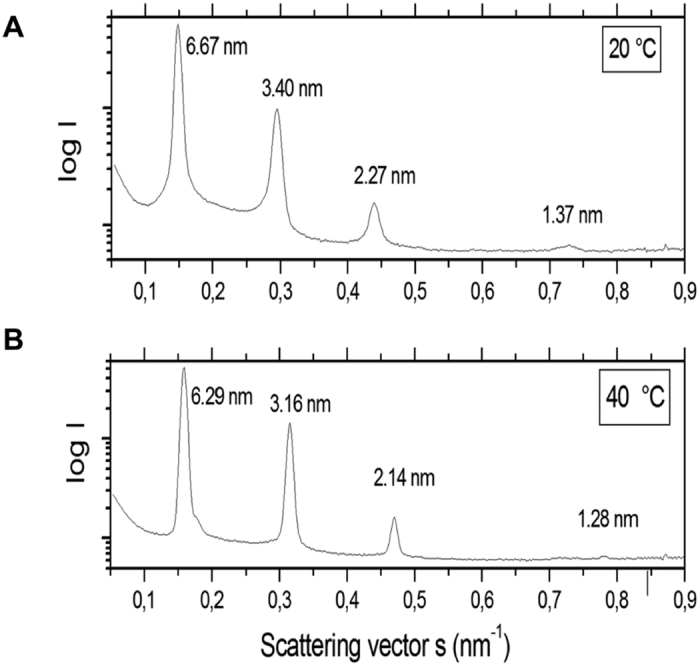
Small-angle X-ray scattering pattern of LPS from *B. quintana* at 20 and 40 °C. The logarithm of the scattering intensity log I is plotted versus the scattering vector s = 1/d (d spacings of the reflections.

To determine the aggregate structure of *B. quintana* LPS, small-angle X-ray scattering (SAXS) at the Hamburg synchrotron source PETRA was applied. For this, LPS at a concentration of 1 mg/50 μl was analyzed at two temperatures 20 and 40 °C (Fig. 9). The scattering patterns are indicative of a main maximum at d = 6.67 and 6.29 nm for 20 and 40 °C, respectively, and further reflections each at d/2, d/3, and d/5, which can be assigned to a multi-lamellar aggregate structure of the LPS dispersion.

## Discussion

Here we described the *in-vitro* and *in-vivo* characteristics of the natural TLR4 antagonist *B. quintana* LPS. LPS of *B. quintana* appears to be a very potent, rapidly binding TLR4 blocker of a potent TLR4 agonist (*E. coli* LPS). In addition, the blockade of TLR4 is prolonged: at least 72 h after exposure to human PBMCs the effect persists. Since TLR4 activation is associated with many inflammatory and autoimmune diseases, *B. quintana* LPS might be considered a new therapeutic strategy for TLR4-driven pathology.

*Bartonella quintana* is an emerging Gram-negative pathogen, which may cause endocarditis, cerebral abscess and bacillary angiomatosis usually with the absence of septic shock in humans. Nowadays, the *B. quintana* infection can be found in homeless people, mainly due to body lice^26^. It has been reported in the past that LPS, isolated from *B. quintana* is able to induce proinflammatory cytokines when injected in rats^27^. This is disagreement with our results, in which highly purified *B. quintana* LPS showed no induction of cytokines, neither at the protein level nor at the level of gene transcription^23^. The main difference between our results and those previously reported by Matera *et al*., is the purity of *B. quintana* LPS. Due to contamination in the crude *B. quintana* extract, predominantly peptidoglycan-driven stimulation of cells through TLR2 ligations may occur. It revealed that Matera *et al*. used only a single isolation step to obtain *B. quintana* LPS (only the first step in our isolation and purification procedure of ultrapure *B. quintana* LPS). It is very likely that this crude preparation of *B. quintana* LPS activates human PBMCs in a TLR2-dependent pathway since peritoneal macrophages obtained from TLR2ko mice did not respond crude *B. quintana* LPS *in vitro,* in contrast to wild type mice (data not shown).

Here we described the structure of *B. quintana* LPS for the first time. It revealed that *B. quintana* LPS has 5 fatty acid tails, two of C12, two of C16 and one very long C26 (Fig. 8). It has been shown previously that LPS structures with 4 fatty acid chains are endotoxically inactive^28^. However, *B. quintana* LPS consists of 5 fatty acids and still is acts as a very potent TLR4 antagonist. This is in line with several other reports demonstrating that LPS originated from Gram-negative bacteria, such as *Bradyrhizobium elkanii* consists of 5 fatty acids tails and reveals to have antagonistic properties^29^,^30^. Studies using small angle X-ray scattering (SAXS) technology indicated that this particular LPS has a multilamellar structure. The data for the aggregate structure of LPS from *B. quintana*, a multilamellar organization, are characteristic for bioinactive structures of endotoxins similar as described by Brandenburg *et al*. and Schromm *et al*.^31^,^32^. In this kind of aggregate structure, the binding epitopes in LPS to the TLR4 receptor necessary for cell signaling are hidden, in contrast to the situation for bioactive LPS with its cubic aggregate structure^31^. However, since the cell activation is a membrane step and also a multilamellar LPS can incorporate into the immune cell membrane, cell receptors such as TLR4 may be blocked by them in this way inhibiting the cell signaling via bioactive LPS. A further observation in accordance to the chemical analysis described here should be mentioned: The periodicities in the range of 6.3 to 6.7 nm as shown in Fig. 9 are characteristic also for multi-lamellar structures of LPS from rough mutant Re and/or Rd from *Salmonella minnesota*, which in a previous report^33^ were found to result from the addition of divalent cations such as Mg^2+^ or at low water content. The final structure of the TLR4 antagonist revealed that the particular molecule is a lipooligosaccharide (LOS) and not a classical lipopolysaccharide (LPS).

Many TLR4 antagonists are based on LPS or lipid A structures obtained from non-pathogenic bacteria such as *Rhodobacter capsulatus* and *Rhodobacter sphaeroides*^34^. Compounds like E5531 (analogue of R. capsulatus lipid A) or Eritoran/E5564 (based on R. sphaeroides lipid A) were developed for the treatment of sepsis. In line with our results, Eritoran is significantly protective in animal models of sepsis^35^. In general, the TLR4 antagonists based on lipid A binds to MD-2 and thereby prevents binding of the agonist to the MD-2/TLR4 complex. This was interpreted to be due to the multilamellar aggregate structure of these antagonists which do not represent a disturbance of the membrane architecture at the site of the receptors, in contrast to the behavior of the non-lamellar aggregate structures of hexaacylated agonistic LPS^36^. Our data show that *B. quintana* LPS has a similar mode of action as E5564. Recently, potent low molecular inhibitors of TLR4 have been reported that interfere with the TLR4-MD2 complex formation^37^.

Polymyxin B is an antibiotic primarily used for resistant Gram-negative infections and it is derived from the bacterium *Bacillus polymyxa*. It has a bactericidal action against almost all Gram-negative bacilli and polymyxin binds to the cell membrane and alters its structure, making it more permeable, resulting in death of the microbe. Polymyxin B is well known for its LPS neutralizing capacity *in vitro*. This can be correlated with the observation, that polymyxin B converts the aggregate structure of agonistic LPS into a multilamellar form^38^. In the case of *B. quintana* LPS, its aggregate structure is already multilamellar, and is not changed furthermore by polymyxin B Furthermore, the fluidization observed when PMB interacts with hexaacylated endotoxins is absent^32^,^38^.

Here we demonstrated that B. quintana LPS binds very rapidly to the TLR4 complex, within 15 minutes the *B. quintana* LPS prevents activation of TLR4. Even 2 h after *E. coli* LPS was added to the cell cultures, *B. quintana* LPS was able to prevent cytokine production. This delayed antagonistic effect was only reported for one other natural TLR4 antagonist, isolated from the cyanobacterium *Oscillatoria planktothrix FP1*^39^. It was demonstrated that the LPS from this cyanobacterium could block DC maturation and activation even 6 h after *E. coli* LPS was added. In line with our report, the cyanobacterium LPS was able to prevent LPS/D-galactosamine induced lethal shock. Although, the dose needed for protection was much higher (750 μg per mouse) than we showed in this current report (100 μg per mouse), indicating the potency of *B. quintana* LPS.

Activation of TLR4 has been associated with many inflammatory diseases and infectious complications^40^,^41^. Therefore, many efforts have been taken to develop or identity potent inhibitors of TLR4 for *in-vivo* applications. Apart from lipid A-derived structures or small molecules that interfere with MD-2/TLR4 formation^42^, antibodies have been developed. However, it seems that anti-TLR4 antibodies do not bind only TLR4, but via the Fc portion also to FcγRs. This dual action of these anti-TLR4 antibodies may be of importance to target inflammatory cells that express both receptors^43^.

Apart from infectious agents that can trigger TLR4 signaling, several endogenous TLR4 ligands have been described in the recent years. Many damage-associated products (DAMP) and inflammatory mediators have been linked to TLR4 for their pro-inflammatory behavior. Most of these TLR4 ligands are released after cells or tissues have been activated or damaged. A few examples of these endogenous TLR4 ligands are HMBG1, S100A7/8, fibronectin extra domain A and fetuin^7^,^44^,^45^,^46^,^47^. Potent inhibitors of TLR4 that interfere with both microbial TLR4 ligands as well as endogenous TLR4 ligands binding to TLR4 will have significant therapeutic value. Since the most TLR4 inhibitors are based on the disruption of the TLR4/MD-2 complex, which is very specific for lipid A-derived compounds, it remains to be explored whether TLR4 antagonist can be generated that block both classes of TLR4 ligands. Of high interest, *B. quintana* LPS reveals to inhibit both exogenous and endogenous TLR4 as previously reported^15^,^48^. Further investigation is warranted to elucidate the structure of *B. quintana* LPS to obtain insight into the mode of action and the possibility to synthesize this potent TLR4 antagonist.

## Materials and Methods

The authors confirm that all experiments were performed in accordance with relevant guidelines and regulations. Written informed consents were obtained from all donors in accordance with the ethical principles set out in the declaration of Helsinki. The ethical review board of the Radboud University Medical Center, Nijmegen, The Netherlands, approved the study in which blood were used for healthy subjects (CMO2299 2010/104). The experimental protocols for murine studies were approved by the ethic committee for animal experiments (DEC) of the Radboud University Medical Center, Nijmegen, The Netherlands.

### Reagents and microorganisms

LPS (*E. coli* serotype O55:B5) was purchased from Sigma Chemical Co and the *Bartonella quintana* CIP 103739 strain was kindly provided by Dr. Tanja Schulin and grown on sheep blood agar at 37 °C in a 5% CO2 atmosphere. *B. quintana* LPS was extracted by a two-step extraction method, which eliminates contamination with proteins. *B. quintana* LPS was extracted by hot phenol-water method as described previously^23^,^49^. Briefly, *Bartonella quintana* bacteria were scraped from blood agar plates, resuspended in PBS and heat-inactivated for 60 min in 56 °C. Thereafter, heat killed bacteria were wash twice with PBS and centrifuged for 10 min. at 16,262 × g. 2 grams of bacterial mass was used to isolate the LPS. Warm water (65 °C) was added to the pellet and the solution was vortexed for 10 minutes. Thereafter, the heated phenol (65 °C) was added and the solution was stirred for 2 hours at a temperature between 63–68 °C. Thereafter, solution was centrifuged 4,435 × g for 40 min at 4 °C. The aqueous phase was collected and transferred to a dialysis cassette (3.500 MWCO) and dialyzed against demi water in a 3L glass beaker in the cold room. The distilled water was changed after 30 minutes for the first time and then after 1 hour for 3–4 times. The LPS was dialyzed for two days at 4 °C, changing demi water 3 times a day. The dialyzed LPS was extracted and stored at −80 °C for until lyophilizing. For re-purification, 5mg *Bartonella quintana* LPS was added to 1 ml 0.2% TEA (Triethylamine)/0.5% Na-DOC (Natrium deoxycholate). Thereafter, 1 ml warm (60 °C) phenol:water (9:1 V/V) was added and the solution was vortexed for 5 min. After separation of the phases (5 minutes at 4 °C) the solution was centrifuged for 40 min. at 6,652 × g (4 °C). The water phase was collected and transferred to new sterile 15 ml tube. To the first phenol phase again 1 ml 0.2% TEA/0.5% Na-DOC was added and the previous steps were repeated. The second phenol phase was used to repeat the purification steps for the third time. The water phase of last 2 steps were combined with the first step. The LPS was dialyzed as described above, using the 3,500 MWCO cassette. To the dialyzed LPS drop-by-drop 1.5 ml of NaAc/EtOH (0.4 M in 100% EtOH) per each 0.5 ml of LPS was added and the solution was kept for 1 h on ice/water to let the LPS precipitate. Thereafter, the LPS was collected by centrifugation (30 min. at 16,262 × g) and washed twice with 1.5 ml cold EtOH followed by centrifugation (30 min. at 16,262 × g). Thereafter, LPS was dried on air, dissolved in PBS, aliquoted and stored by −20 °C. *E. coli* LPS from Sigma was also double purified, as described above.

### Isolation of PBMC and stimulation of cytokine production

Peripheral blood mononuclear cells (PBMCs) were isolated healthy individuals (written informed consent was obtained from all subjects), as described earlier^50^,^51^. Briefly, PBMCs were isolated by density gradient centrifugation using Ficoll-Paque PLUS (GE Healthcare) and collecting the white interphase. Next, PBMCs were washed twice in cold PBS and concentrations were adjusted to 5 × 10^6^ cells/ml in RPMI-1640 Dutch Modified culture medium (RPMI supplemented with 2 mM l-glutamine, 1 mM pyruvate; GIBCO Invitrogen, Carlsbad, CA, USA). PBMC (5 × 10^5^), in a volume of 100 μl volume in round-bottomed 96-well plates (Greiner, Alphen a/d Rijn, The Netherlands), were incubated with either 100 μl of culture medium (negative control) or one of the following stimuli: *B. quintana* LPS and *E. coli* LPS (10 ng/ml).

### Measurement of cytokine concentrations

Cytokines were determined by commercially available ELISA kits according to manufacturer’s instructions. Concentrations of human IL-1β, TNF-α, (R&D Systems, Inc., Minneapolis, MN, USA), IL-6 and IL-8 (Sanquin Reagents, Amsterdam, The Netherlands) were measured^52^,^53^.

### Animals

C57Bl/6J mice were purchased from Charles River (Sulzfeld, Germany). For the experiments, 8–12 week old mice, weighing 20–25 g, were used. The animals were fed standard laboratory chow (Hope Farms, Woerden, The Netherlands) and housed under specific pathogen-free conditions. The experimental protocols were approved by the ethic committee for animal experiments (DEC) of the Radboud University Medical Center, Nijmegen, The Netherlands.

### Experimental endotoxaemia model

The previously reported model of endotoxemia was used^54^. Briefly, 20 wild type mice were injected intraperitoneally (i.p.) with either PBS, or *B. quintana* LPS 100 μg/mouse. After 30 minutes 10 mice of each group were injected with PBS and 10 mice were injected i.p. *E. coli* LPS 1 μg per mouse (Escherichia coli LPS 055:B5 Sigma Chemical Co., St Louis, MI, USA) + D-galactosamine 14 mg per mouse. Survival of all 4 groups (PBS + PBS, *B. quintana* LPS + PBS, PBS + *E. coli* LPS/D-galactosamine and *B. quintana* LPS + *E. coli* LPS/D-galactosamine was assessed for 10 days.

### Structural analysis of *B. quintana* LPS

The compositional analysis was done by using combined gas liquid chromatography and mass spectrometry (GLC-MS), as well as electrospray ionization mass spectrometry (ESI-MS). For the GLC-MS the *B. quintana* LPS was methanolyzed by 2 M HCL/CH3OH for 24 h at 85 °C and for the determination of the hexoses afterwards peracetylated or trimethylsilylated with N,O-bis(trimethylsilyl)trifluoroacetamide for the fatty acids, respectively. The resulting compounds were analyzed in a GLC on a Hewlett-Packard HP 5890 Series II chromatograph, equipped with a 30-m fused silica SPB-5 column (Supelco) using a temperature gradient of 150 °C (3 min) → 320 °C at 5 °C/min, and GLC-MS on a Hewlett-Packard HP 5989A instrument equipped with a 30-m HP-5MS column. Electrospray Ionization Fourier Transform Ion Cyclotron Resonance (ESI FT-ICR) MS was performed in using a hybrid Apex Qe FT-ICR MS instrument (Bruker Daltonics) in the negative ion mode, equipped with a 7 Tesla actively shielded magnet and an Apollo dual ion source^55^. Small-angle X-ray scattering (SAXS) measurements of LPS from *Bartonella quintana* were performed at the European Molecular Biology Laboratory outstation at the Hamburg synchrotron radiation facility (HASYLAB) using the double-focusing monochromator-mirror camera X33. Scattering patterns in the range of the scattering vector 0.01 < s < 1 nm^−1^ (s = 2 sinθ/λ, 2θ = scattering angle, λ = wavelength = 0.15 nm) were recorded at 20 and 40 °C with exposure times of 1 min using an image plate detector with online readout (MAR345; MarResearch, Norderstedt/Germany)^56^. The s-axis was calibrated with Ag-Behenate, which has a periodicity of 5.84 nm. We evaluated the diffraction by assigning the spacing ratios of the main scattering maxima to defined three-dimensional structures. For this study, the multi-lamellar structures were the most relevant, for which characteristic spacing’s at the periodicity d and further reflections at d/2, d/3 etc. are found.

### Statistical analysis

The data are expressed as mean ± SEM. Differences between experimental groups were tested using the two-sided Mann-Whitney *U* test or one-way ANOVA performed on GraphPad Prism 6.0 software (GraphPad). *P* values of ≤0.05 were considered significant. The IC50 was calculated using non-linear concentration-response curve application within GraphPad Prism 6.0.

## Additional Information

**How to cite this article**: Malgorzata-Miller, G. *et al*. *Bartonella quintana* lipopolysaccharide (LPS): structure and characteristics of a potent TLR4 antagonist for *in-vitro* and *in-vivo* applications. *Sci. Rep.* **6**, 34221; doi: 10.1038/srep34221 (2016).
